# Exploratory, Phase II Controlled Trial of Shiunko Ointment Local Application Twice a Day for 4 Weeks in Ethiopian Patients with Localized Cutaneous Leishmaniasis

**DOI:** 10.1155/2016/5984709

**Published:** 2016-04-19

**Authors:** Kesara Na-Bangchang, Oumer Ahmed, Jemal Hussein, Kenji Hirayama, Panida Kongjam, Abraham Aseffa, Juntra Karbwang

**Affiliations:** ^1^Graduate Studies, Chulabhorn International College of Medicine, Center of Excellence in Pharmacology and Molecular Biology of Malaria and Cholangiocarcinoma, Thammasat University, Pathum Thani 12121, Thailand; ^2^Armauer Hansen Research Institute (AHRI), 1000 Addis Ababa, Ethiopia; ^3^Department of Immunogenetics, Institute of Tropical Medicine (NEKKEN), Graduate School of Biomedical Sciences, Nagasaki University, Nagasaki 852-8523, Japan; ^4^Clinical Product Development, Institute of Tropical Medicine (NEKKEN), Graduate School of Biomedical Sciences, Nagasaki University, Nagasaki 852-8523, Japan

## Abstract

The clinical efficacy and safety of Shiunko ointment (phase II clinical trial) was investigated in 40 Ethiopian patients with cutaneous leishmaniasis. Patients were randomized to receive treatment with Shiunko ointment or placebo (*n* = 20, each), applied on the lesion twice a day for 4 weeks. Clinicoparasitological assessments were performed before treatment, weekly for 4 weeks, and then 4, 8, and 12 weeks after the end of treatment. A marked reduction in lesion size was observed on week 16 of treatment in the Shiunko compared with placebo group (69% and 22% reduction, resp.). The overall rate of lesion reduction during the four weeks of treatment was significantly faster in the Shiunko group. Shiunko provided significant effect on wound closure in patients with ulcerated lesion. The clinical efficacy and tolerability of Shiunko were comparable to placebo with regard to its clinicoparasitological response (cure rate and parasitological clearance). Results of this preliminary study may suggest that Shiunko could be useful as adjuvant or as complementary treatment, not as alternatives to current treatment. Its attractive action includes fast lesion healing with a significantly smaller lesion at week 16 of treatment compared with placebo. In addition, its action was promoted in ulcerative lesions.

## 1. Introduction

Cutaneous leishmaniasis (CL) is a major tropical skin disease which represents a worldwide public health and social problem in many developing countries including Ethiopia. There are approximately 1.5 million new cases of CL each year, of which more than 90% occur in Afghanistan, Iran, Saudi Arabia, Syria, Brazil, and Peru [1]. CL in Ethiopia is predominantly caused by* Leishmania aethiopica* (99%) and rarely due to* Leishmania major* and* Leishmania tropica* [2]. The clinical manifestation of* L. aethiopica* infection is mostly in a localized form (LCL), while the diffuse and mucocutaneous forms are rare [3–5]. However, a recent report [6] showed a relatively higher percentage (19.2%) of mucocutaneous CL due to* L. aethiopica* in the Silte, Ethiopia, outbreak. The primary lesion in CL patients may be a patch of erythematous induration at the site of sandfly bite that may progress to papulonodular, plaque, or ulcerative lesion. Many lesions do not ulcerate but persist as nodules or plaques. When it ulcerates, it forms a central depression and raised indurated border. Nodules plaques or ulcers may enlarge to a diameter of several centimeters. LCL may persist for months or years before eventually healing with an atrophic scar.

The treatments available for CL include cryotherapy and intralesional injection of sodium stibogluconate. The cure rate from these treatments varies and has been claimed to be between 60 and 70% [7]. However, none of the treatments currently used in Ethiopia provide a solid evidence to support the development of a national treatment guideline for CL [7]. This is further complicated by a significant coinfection of HIV and CL [8]. The incidence of CL continues to increase, and disease control and management of CL in Ethiopia remains a challenge. New safer, improved efficacious treatment(s) is urgently needed. One such new candidate is Shiunko, a Chinese medicinal ointment that was formulated by the innovative Japanese surgeon Seishu Hanaoka in the Edo era (1603 to 1868). It is composed of Lithospermi Radix (shikon), the root of* Lithospermum erythrorhizon*; Angelicae Radix (tohki), the root of* Angelica acutiloba* Kitagawa; sesame oil; and honeycomb wax. The medicine is widely available in Japan as an “Over-the-Counter (OTC)” product for burn lesions, bedsore, eczema dermatitis, and hemorrhoid anal fissure indications. Shiunko ointment has been demonstrated to possess anti-inflammatory, analgesic, hemostatic, and antiseptic properties in various studies [9]. The antileishmanial activity of Shiunko was discovered in 1984 by the Japanese scientists with financial support from the Japan Health Sciences Foundation (JHSF), Tokyo [10]. In the first study, clinical efficacy of topical Shiunko ointment twice daily for 4 weeks was conducted in 26 patients with Peruvian LCL (*L. mexicana*). Of the 14 patients who completed the follow-up period (12 weeks), treatment response in 3 (21.4%) patients was significantly effective (almost completely recovered or cured); treatment responses in 8 cases (57.1%), 2 cases (14.3%), and 1 case (7.2%), respectively, were considered sufficiently effective (obviously recovered), effective (slightly recovered), and not effective (not recovered or remaining unchanged). The trial was continued to recruit more LCL patients to a total of 53 cases. A promising result was obtained in all cases who were selected based on resistance to systemic drug administration. Forty-six (86.8%) patients had complete cure after local administration of Shiunko for 4 weeks, with complete absence of parasites in lesion biopsy at the end of treatment. The aim of the present study was therefore to evaluate the clinical efficacy and safety of Shiunko ointment when applied on the lesion twice a day for 4 weeks in Ethiopian patients with LCL.

## 2. Materials and Methods

### 2.1. Patients and Study Design

The study was an exploratory phase II, placebo-controlled, single-center (Ankober Health Center, North Shewa zone of Amhara region, Ethiopia) study. Patients with localized cutaneous leishmaniasis (LCL) aged 18–65 years of both genders with a new, uncomplicated, localized, single lesion on the face or arms and positive parasite smear by microscopy were included in the study. Patient's exclusion criteria were (i) cutaneous leishmaniasis with secondary infection, (ii) concomitant diseases (mucocutaneous or visceral leishmaniasis), (iii) history of antileishmanial treatment within the past six months, (iv) abnormality of biochemical and/or hematological laboratory tests, (v) known hypersensitivity to any of the Shiunko components, or (vi) pregnancy (positive urine HCG test), breast-feeding, or possibility of becoming pregnant during the study. All patients had residential areas in North Shewa zone of Amhara region (Chefa site, Derefo Nefasso, Derefo Nadewa, Gorebela Chaka, Gorebela Zembo, Aleyu Amba, Debre Hayl, Haramba, Deway Derefo, Wubit Gola, Mehal Wonz, Lay Gorebela, and Metak), Ethiopia. These are the high transmission areas which constitute the main LCL endemic area in the country, contributing over 60% of the burden.

Lesion sample was collected from each patient by slit-skin scraping from the lesion and diagnosis of CL was based on microscopic examination for the presence of leishmanial amastigotes. Written informed consents for study participation were obtained from all of them before study. The study was approved by the Ethics Committees of Armauer Hansen Research Institute (AHRI), Ethiopia, and the Institute of Tropical Medicine, Graduate School of Biomedical Sciences, Nagasaki University, Japan (UMIN Clinical Trials Registry: UMIN-CTR Number 000010994).

### 2.2. Treatment

Patients who fulfilled inclusion and had none of the exclusion criteria were randomized to receive Shiunko ointment and placebo (20 patients* per *group). Preparation of the pharmaceutical formulations of Shiunko (batch number 961) and the placebo (batch number 962) including quality assurance and control were performed by Okusa Co., Ltd. (Japan). Both were prepared as an ointment pharmaceutical formulation (20 g of violet red ointment* per *container). The composition of Shiunko was as follows: 2.04 g shikon, 1.02 g tohki, 16.92 g sesame oil, and 6.76 g honeycomb wax. The placebo contained 20 g wax. The ointment was applied locally by the health post extension workers, covering the whole lesion of each patient twice a day (in the morning and evening, 8–10 hours apart), for 4 weeks. All antileishmanial drugs were prohibited during the study period. Cryotherapy was to be offered to all patients who were withdrawn or discontinued from the study for safety reasons (i.e., those with serious adverse events or with increase of lesion size by more than 50% of the original size, or treatment failure). Patients who withdrew their consents were evaluated by the dermatologist for the requirement of rescue treatment.

### 2.3. Assessment of Efficacy and Safety

#### 2.3.1. Clinical Assessment

The lesion was observed for the progress of reepithelization before treatment (on recruitment) and weekly for 4 weeks (during treatment) and then 4, 8, and 12 weeks after the end of treatment. Each lesion was photographed to document the appearance of the lesion until declared cured. The rate of lesion size reduction was estimated from the slope of each of the average lines of the lesion areas reduction from week 0 up to week 4 of treatment.

#### 2.3.2. Parasitological Assessment

Lesion sample was taken from margin of the ulcerated lesion after removal of the crust and cleaning of the lesion by scraping (for open wounds) or by fine needle aspiration before treatment, at the end of treatment and monthly until parasitological negativity, that is, absence of amastigotes in the smear. Parasitological smear (Giemsa staining) was examined by two different parasitologists (AHRI laboratory), under light microscope (×100) for the presence or absence of leishmanial amastigotes. In addition, all samples were confirmed by an external expert (Institute of Tropical Medicine: NEKKEN).

*Safety Assessment*. Safety and tolerability of Shiunko and placebo were assessed based on clinical and laboratory assessments during follow-up, according to the NIH/NCI Common Toxicity Criteria (CTC) grading system [11].

### 2.4. Data Analysis

Treatment efficacy of Shiunko and placebo was categorized as cure, partial response, and treatment failure. Cure was defined as complete wound closure and reepithelization without inflammation or infiltration within 12 weeks after the end of treatment and absence of parasite (amastigotes) within 12 weeks after the end of treatment. Partial response was defined as improvement of* Leishmania* signs (skin edema, erythematous, and/or hardening) and/or reduction in size but not total disappearance of the lesion within 12 weeks after the end of treatment and absence of parasite (amastigotes) within 12 weeks after the end of treatment. Treatment failure was defined as failure of the lesion size to decrease and/or lack of lesion sign improvement or reepithelization and/or presence of leishmanial amastigotes in the lesion 12 weeks after end of treatment (week 16) [12].

The primary efficacy analysis was based on intention-to-treat (ITT) population (inclusion of all patients as originally allocated after randomization) with secondary analysis of per-protocol (PP) population. PP population was a subset of the subjects in the full analysis set who were more compliant with the protocol and was characterized by the following criteria: (i) the completion of at least 85% of the treatment regimen; (ii) the availability of measurements of the primary variable(s); and (iii) the absence of any major protocol violations including the violation of entry criteria.

Treatment safety was evaluated based on the proportions of patients with adverse events (AE) including serious adverse events (SAE) following treatment. These included local (pain, erythema, induration, swelling, itching, nodules, etc.) and systemic (diffuse erythema, urticaria, fever, headache, anaphylactic reactions, etc.) adverse events, as well as clinical relevant changes in laboratory parameters. The analysis was based on ITT population. The proportion of patients experiencing any adverse event during the 16-week study period was evaluated.

Quantitative data are presented as median (range and 95% CI) and qualitative data are presented as number and proportion (%) with 95% CI. Comparison of quantitative data of each study period and baseline was performed using Wilcoxon Signed Rank test. Comparison of quantitative data between the two groups during each study visit was performed using Mann-Whitney *U* test. Comparison of qualitative data was performed using Chi-square test. Statistical significance was set at *α* = 0.05 for all tests (SPSS version 16.0, SPSS Inc., CO, USA).

## 3. Results

### 3.1. Patients

Out of 108 patients screened (61 males and 47 females), 40 patients with CL (25 males and 15 females, aged 18–62 years) who fulfilled inclusion and had none of the exclusion criteria were randomized to receive placebo and Shiunko treatment (20 patients for each group). The most common locations for CL lesions were the face (80%), followed by arms (10%), neck (7.5%), and eyelids (2.5%). The most common signs and symptoms in both groups of patients were pruritus or itching sensation (65%), pain (32.5%), burning sensation (17.5%), headache (10%), eye discharge (2.5%), abdominal distension (2.5%), myalgia (2.5%), and paraesthesia (2.5%). Demographics and baseline lesion size (Table 1) including laboratory data (hematology and biochemistry) of patients in both groups were comparable.

### 3.2. Efficacy Evaluation

**Figure 1:**
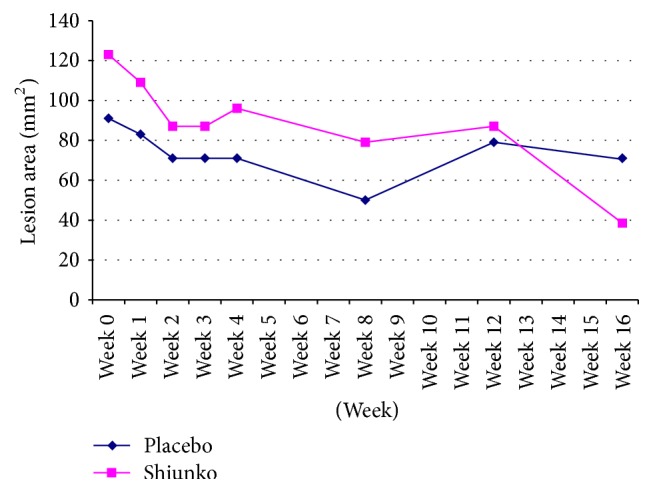
Lesion progression (lesion size: mm^2^) observed during treatment and follow-up periods in Shiunko and placebo groups. The rate of lesion reduction during treatment period in the Shiunko group was significantly higher than placebo group (Wilcoxon Signed Rank test, *p* = 0.043).

**Figure 2:**
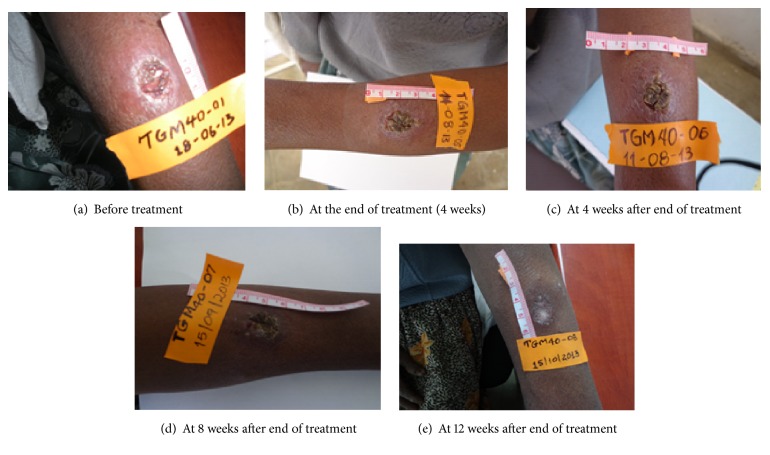
Representative photos of the lesions in a patient with cure following treatment with Shiunko ointment: (a) before treatment, (b) at the end of treatment, (c) at 4 weeks after end of treatment, (d) at 8 weeks, and (e) at 12 weeks of treatment.

Following the start of treatment with Shiunko and placebo, lesion size gradually reduced until the end of follow-up (week 16) (Figure 1). The lesion size on week 16 was significantly reduced compared with baseline (*p* = 0.03) in Shiunko group.  Figure 2 shows representative photos of the lesions in a patient with cure after treatment with Shiunko ointment (before treatment, at the end of treatment, and 4, 8, and 12 weeks after the end of treatment). The median lesion size on week 16 was about 31% of baseline in Shiunko group, while it was 78% in placebo group. In addition, the overall rate of lesion reduction during the four-week treatment period was significantly faster in the Shiunko compared with placebo (*p* = 0.043). A total of 178 parasitological smears collected before treatment, at the end of treatment, and at 4, 8, and 12 weeks after treatment were confirmed by an expert at NEKKEN laboratory. Discrepancy of results was found only in four slides (2.25%) collected from four patients on different occasions, that is, 1 slide at 4 weeks from Shiunko group, 1 slide at 8 weeks from placebo group, and 2 slides at 12 weeks (one slide each for Shiunko and placebo) after treatment. All were initially reported as negative but were finally confirmed as positive. Lesion healing occurred 2 weeks after treatment in both groups of patients. The patterns and proportions of patients with* Leishmania* signs (edema, erythema, inflammation, nodule, etc.) in both groups during treatment and follow-up were comparable. There were no significant relationships between treatment response and location of lesion, treatment response and lesion size, parasite load and lesion size, or lesion size and wound closure. A significant association was observed between lesion ulceration and treatment response in Shiunko group (*p* = 0.03). The proportion of patients with satisfactory treatment response (complete cure and/or partial response) who had ulcerated lesions was significantly higher than those with treatment failure who had no ulcerated lesion (Table 2).

**Figure 3:**
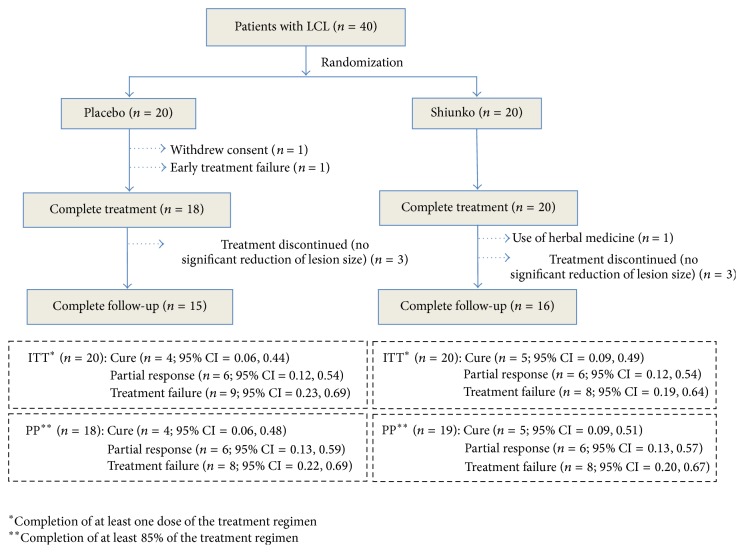
Efficacy assessment (based on ITT and PP analysis) of CL patients following treatment with Shiunko and placebo.

**Figure 4:**
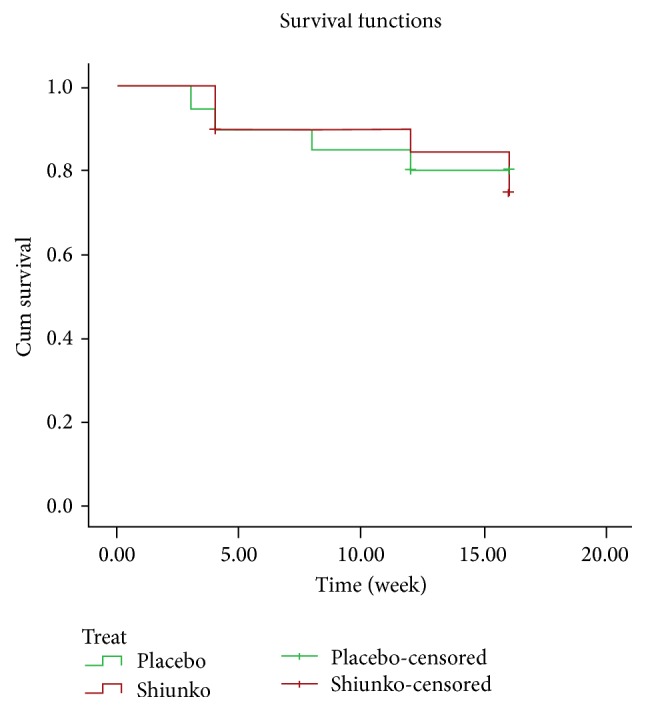
Kaplan-Meir survival curves in cutaneous* Leishmania* patients following the 16-week observation period in Shiunko and placebo groups.

Thirty-eight (95%) and 31 (77.5%) patients had complete treatment and complete follow-up, respectively. For the placebo group, 18 (90%) and 15 (75%) cases had complete treatment and follow-up, respectively. One patient withdrew consent for study participation during treatment and one had early treatment failure. Three patients discontinued the study during 8- and 16-week follow-up due to deterioration of lesion and/or increase in lesion size by 50% of baseline. For the Shiunko group, 20 (100%) and 16 (80%) had complete treatment and follow-up, respectively. One and three patients, respectively, discontinued the study during 8- and 16-week follow-up due to herbal medicine usage and deterioration of lesion and/or lesion size by 50% of baseline. Clinical efficacy of Shiunko versus placebo based on ITT and PP analysis was similar with regard to proportions of patients with cure, that is, 20% (*n* = 4) versus 25% (*n* = 5) and 22.2% (*n* = 4) versus 26.3% (*n* = 5)%, respectively (Figure 3).  Figure 4 shows Kaplan-Meir survival curves in both groups; the number of patients with satisfactory response was comparable in both groups. The probability of cure at 4, 8, and 12 weeks after treatment with Shiunko and placebo was similar.

### 3.3. Safety Evaluation

**Figure 5:**
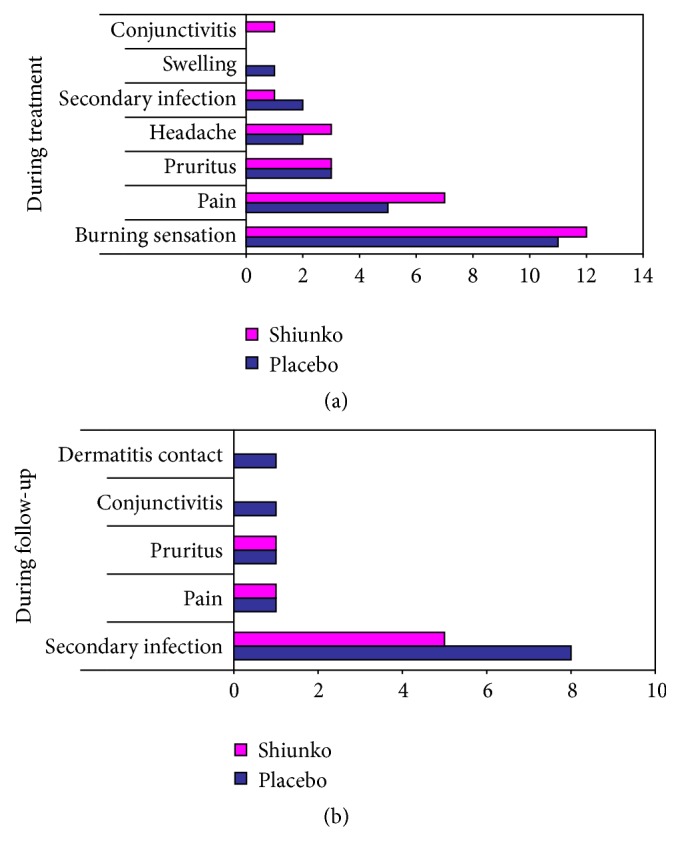
Frequencies (number) of adverse events observed during (a) treatment and (b) follow-up periods in Shiunko and placebo groups.

There was no significant change in most laboratory parameters (hematology and biochemistry) in both groups of patients after treatment compared to baseline. However, significant reduction in serum AST was observed in the placebo group (245 IU/L), while significant reduction in creatinine (43 mol/L) and increase in neutrophil (57%) were observed in the Shiunko group. The frequencies of adverse events in descending order in both groups of patients during treatment were burning sensation, followed by pain, pruritus (itching sensation), headache, secondary infection, swelling, and conjunctivitis (Figure 5). The corresponding frequencies during the follow-up periods were secondary infection, pain, pruritus, conjunctivitis, and dermatitis contact (Figure 5). The intensity of all adverse events was mild to moderate and was nonserious. Burning sensation pruritus and pain were possibly related to the study medication (Shiunko and placebo). The frequencies of all adverse events were comparable between the two groups. Secondary infection occurred as early as 7 days until 16 weeks of treatment but in most cases was resolved within the follow-up period.

## 4. Discussion

Results from this exploratory study suggest the potential of Shiunko ointment as a treatment of LCL. This is supported by its marked reduction of the lesion size with a significantly faster rate during the four-week treatment period in the Shiunko compared with placebo group (Figure 1), as well as its safety profile. Furthermore, Shiunko treatment provided notable therapeutic effect on wound closure in patients with ulcerated lesion. Patients with complete cure had a significantly higher ratio of lesion ulceration (the ratio of cases with and without ulceration = 3 : 2) compared with those with partial (1 : 4) and treatment failure (0 : 9). This suggests that Shiunko treatment may provide more beneficial effect in patients with ulcerated lesions. As a result of the damage of skin barrier, penetration of the active compounds from Shiunko ointment to leishmanial parasite located inside the wound could be improved. It was noted however that the final assessment endpoint was based on parasitological negativity which was the absence of amastigotes in the smear. The treatment was well tolerated with only mild-to-moderate nonclinically significant adverse events and changes in laboratory parameters. Burning sensation, pruritus, and pain were possibly related to the ingredients in the study medication as they were found in both groups. The side effects documented in the Shiunko ointment package insert include rash and pruritus. The safety of Shiunko is not a concern when it is applied locally as it has been used extensively in Japan for bedsore lesion and classified as OTC medication. Its categorization as an OCT medication suggests that it has a high safety profile and can be used without doctor's prescription.

The advantage of Shiunko treatment over placebo could not have been clearly demonstrated in this exploratory study due to limitation of the study design and sample size. The clinical efficacy of Shiunko in the current dosage regimen and application procedure was shown to be comparable to placebo with regard to its clinicoparasitological response (cure rate and parasitological clearance). One of the weaknesses of the study is that the duration of the lesion prior to enrolment was not well controlled and thus natural healing could have occurred in those with older lesions. In this study, patients with new lesions of shorter than 6 months (5–22 weeks) were included in the study. Only patients with lesion of shorter than three or six months in duration (depending on the species) should have been included in the study. Although the study was randomized, the possibility of enrolling patients with older lesion may not have been equally distributed between the two treatment arms, and this may have swayed the healing rate of the lesion. In addition, the recruited patients presented with different types of lesions which may require different healing processes. There were twelve patients with partial response (negative parasite but the healing of the lesion was not completed). This observation may suggest that the follow-up period may not be sufficient for the completion of healing process in these patients. Unfortunately, the leishmanial species could not be confirmed in this study due to problem related to the quality of the PCR samples.

The clinical efficacy of Shiunko in the current dosage regimen and method of application was considered low when compared with that previously reported in Peruvian* L. mexicana* (LCL). The initial exploratory study demonstrated high clinical efficacy of Shiunko ointment when administered locally twice a day for 4 weeks in 14 patients with localized CL [10]. Satisfactory treatment response (almost completely cured, obviously cured, and slightly cured) was observed in almost all cases (13 cases) in the initial study, and 46 of 63 (86.8%) patients with complete cure were observed in the following study. The obviously low clinical efficacy of Shiunko ointment (26.3% complete cure based on PP analysis) observed in our group of patients could be explained mainly by difference in sensitivity of parasite species. Furthermore, it has been reported that different leishmanial species reside in different macrophage types with different adaptations that facilitate intracellular survival [13, 14]. Such species variation accounts for differences in parasites' susceptibility to drugs [15]. The effect of Shiunko treatment could have also been clearly demonstrated with prolonged follow-up period beyond 16 weeks (Figure 1).

Shikonin has been reported to be the principal component of Shiunko ointment. It is derived from a traditional Chinese herbal medicine “Zicao” which is prepared from the dried root of* Lithospermum erythrorhizon*. Reports from various studies in recent years have provided a scientific basis for the clinical use of this medicine for a variety of inflammatory and infectious diseases. Diverse biological and pharmacological properties of shikonin, the active component of Shiunko, have also been well demonstrated in various* in vitro* and animal models. These include antioxidant, anti-inflammatory, antimicrobial, anticancer, and wound healing properties. The wound healing effect of shikonin/alkannin-based ointment was shown in dogs to be due to its effect on promoting angiogenesis, collagen production, and epithelialization of the lesions [16]. Furthermore, the molecular basis of wound healing process of the ethanol extract of the root of* L. erythrorhizon *and shikonin was suggested to be through a synergistic effect on a number of cell processes including antioxidation, antiapoptosis, cell mobility, cell proliferation, metabolism of abnormal proteins, and collagen secretion [17, 18]. Upregulation of several proteins associated with proliferation and antiapoptosis of the treated fibroblast cells was demonstrated [18]. With respect to the biopharmaceutical properties, the active principal shikonin is highly lipophilic and water-insoluble, which supports its uses as creams and ointments formulations [19]. The wide spectrum of activities, biological properties, and molecular targets make shikonin-based Shiunko ointment a treatment of great advantage in CL. The free radical scavenging properties and anti-inflammatory activities of shikonin [20] would be expected to enhance the clinical efficacy of Shiunko in the topical or transdermal formulations. The antimicrobial activity of shikonin is an added benefit to its wound healing properties since a drug that can close a wound while preventing infection and swelling is highly beneficial.

Since the result of this exploratory study is inconclusive, it may not provide definite conclusion on the efficacy of Shiunko ointment for treatment of LCL. However, information obtained may suggest its potential role as an adjuvant or as a complementary treatment to standard treatment. Its attractive action includes fast lesion healing with a significantly smaller lesion at week 16 of treatment compared with placebo. In addition, its action was promoted in ulcerative lesions. Further study should be carried out to confirm its clinical efficacy in a larger number of patients with confirmed identification of* Leishmania* species and well-defined clinical signs and symptoms of CL and with prolonged treatment period and follow-up. In addition, modification of the method of treatment application (e.g., sustained topical application of ointment instead of twice daily administration) is required to allow sustainable drug exposure of the infective lesions and improvement of drug absorption across the skin barrier. An experiment in a mouse model of leishmaniasis with dermatological, histological, and parasitological assessments should be performed before implementation of the phase III study.

## Figures and Tables

**Table 1:** The demographic and baseline clinical parameters before treatment in 40 CL patients allocated to receive treatment with Shiunko and placebo.

Parameters	Placebo (*n* = 20)	Shiunko (*n* = 20)
Age [median (range), years]	40.4 (18–53)	38.1 (18–62)
Sex [number of males and females]	12, 8	13, 7
Lesion sign		
Lesion size [median (range), mm^2^]	91 (16–1,575)	123 (13–1,578)
Lesion location [*n* (%)]		
Face	17 (85)	15 (75)
Neck	2 (10)	1 (5)
Arm	1 (5)	3 (15)
Eyelid	0 (0)	1 (5)

**Table 2:** The proportions of patients with satisfactory treatment response (complete cure and/or partial response) and treatment failure with lesion ulceration in Shiunko and placebo (*n* = 4 and 8 cases, resp.) groups (significantly different by Chi-square test, *p* = 0.03).

	Shiunko *n* (%)	Placebo *n* (%)
Cure	3 (75%)	3 (38%)
Partial response	1 (25%)	1 (13%)
Failure	0 (0%)	4 (49%)
*Total*	*4*	*8*
